# High prevalence of albuminuria amongst people who inject drugs: A cross-sectional study

**DOI:** 10.1038/s41598-020-63748-4

**Published:** 2020-04-27

**Authors:** C. R. McGowan, T. Wright, D. Nitsch, D. Lewer, R. Brathwaite, J. Scott, V. Hope, D. Ciccarone, J. Dunn, J. Gillmore, A. Story, M. Harris

**Affiliations:** 1 Department of Public Health, Environments & Society, Faculty of Public Health & Policy, London School of Hygiene & Tropical Medicine, 15-17 Tavistock Place, London, WC1H 9SH UK; 2 Humanitarian Public Health Technical Unit, Save the Children UK, 1 St John’s Lane, London, EC1M 4AR UK; 3 Department of Non-communicable Disease Epidemiology, Epidemiology & Population Health, London School of Hygiene & Tropical Medicine, Keppel Street, London, WC1E 7HT UK; 4 UCL Collaborative Centre for Inclusion Health, Institute of Epidemiology and Health Care University College London, 1-19 Torrington Place, London, WC1E 7HB UK; 5 Department of Social Genetic & Developmental Psychiatry, Institute of Psychiatry, King’s College London, 16 De Crespigny Park, London, SE5 8AF UK; 6 Department of Pharmacy and Pharmacology, University of Bath, Claverton Down, Bath, BA2 7AY UK; 7 Public Health Institute, Liverpool John Moores University, 79 Tithebarn Street, Liverpool, L2 2ER UK; 8 University of California, San Francisco, Department of Family and Community Medicine, 500 Parnassus Avenue, San Francisco, CA 94143 United States; 9 Camden & Islington NHS Foundation Trust, 108 Hampstead Road, London, NW1 2LS UK; 10 National Amyloidosis Centre, Centre for Amyloidosis & Acute Phase Proteins, Division of Medicine, University College London, Rowland Hill Street, London, NW3 2PF UK; 11 University College Hospitals NHS Foundation Trust, 250 Euston Road, London, NW1 2PG UK

**Keywords:** Chronic kidney disease, Predictive markers, Cardiovascular diseases, Risk factors

## Abstract

Albuminuria is a key biomarker for cardiovascular disease and chronic kidney disease. Our study aimed to describe the prevalence of albuminuria amongst people who inject drugs in London and to test any potential associations with demographic characteristics, past diagnoses, and drug preparation and administration practices. We carried out a cross-sectional survey amongst people who use drugs in London. The main outcome measure was any albuminuria including both microalbuminuria and macroalbuminuria. Three-hundred and sixteen samples were tested by local laboratory services. Our study initially employed point-of-care testing methods but this resulted in a high number of false positives. Our findings suggest the prevalence of albuminuria amongst PWID is twice that of the general population at 19% (95%CI 15.3–24.0%). Risk factors associated with albuminuria were HIV (aOR 4.11 [95% CI 1.37–12.38]); followed by overuse of acidifier for dissolving brown heroin prior to injection (aOR 2.10 [95% CI 1.04–4.22]). Albuminuria is high amongst people who inject drugs compared to the general population suggesting the presence of increased cardiovascular and renal pathologies. This is the first study to demonstrate an association with acidifier overuse. Dehydration may be common amongst this population and may affect the diagnostic accuracy of point-of-care testing for albuminuria.

## Introduction

People who inject drugs (PWID) may be at risk of serious cardiovascular and kidney diseases, including AA amyloidosis, related to chronic inflammatory conditions and poor overall health. Certain drug injecting practices (e.g. subcutaneous injecting, reuse of injecting equipment) are associated with chronic or recurrent abscesses and other skin and soft tissue infections (SSTIs)^1^. Currently, there are few studies looking at early risk markers of cardiovascular disease and chronic kidney disease among PWID, though there is some evidence to suggest that PWID are at increased risk of developing end-stage renal disease^2–5^. Other injecting practices believed to precipitate inflammation relate to the over-use of acidifiers used to dissolve brown heroin which is the predominant type of heroin available in Europe^6^. It is well established that chronic inflammation is associated with both cardiovascular disease and chronic kidney disease^7,8^.

Albuminuria is one of the two biochemical markers used to identify the presence of both cardiovascular disease and chronic kidney disease^9^. The presence of albuminuria is a key risk marker for predicting progression to end-stage renal disease, and for identifying other serious sequelae including AA amyloidosis^9^. A person with elevated albuminuria but with normal estimated glomerular filtration rate (eGFR) has the same cardiovascular risk as a person with an low eGFR (<60) without albuminuria^9,10^. Albuminuria has been associated with additional microvascular pathologies (e.g. involving the skin, brain, lungs) which suggests that albuminuria may also be a marker of systemic microvascular disorder^11^. Albuminuria is associated with cardiovascular risk factors including; overweight/obesity, diabetes, high blood pressure, and tobacco smoking^10^.

Population-level estimates reveal a high prevalence of albuminuria in the UK. A 2004 study among those aged 40–79 years in Norfolk, UK estimated the prevalence of microalbuminuria as 11.2% (13.9% in women, 8.1% in men) and macroalbuminuria as 0.7% (0.7% in women, 0.8% in men)^12^. The 2009 and 2010 Health Surveys for England estimated that 8% of adults surveyed had abnormal levels of albumin in their urine (7.9% microalbuminuria, 0.5% macroalbuminuria)^10^.

This paper aims to describe the prevalence of albuminuria among PWID, and to inform the clinical use and interpretation of albuminuria testing amongst this population.

## Methods

We undertook a cross-sectional survey, as part of the UK National Institute for Health Research-funded Care & Prevent study, aimed at assessing the evidence for AA amyloidosis amongst PWID^13^. We developed a computer-assisted questionnaire to identify patterns of drug use and potential risk factors for SSTIs and AA amyloidosis. The questionnaire was conducted with current or past PWID aged 18 years and over who were recruited in London at eight drug treatment services and a mobile health service working with homeless people (UCLH Find & Treat Service). Participants completed the questionnaire followed by a urine screen for albuminuria. Participants were asked questions relating to: demographics, injecting history, injection practices, HIV and hepatitis C virus (HCV) status, SSTI history, and other conditions associated with albuminuria. Potential confounders were age, sex, and tobacco smoking.

Urine was initially tested using laboratory urinalysis and/or point of care (POC) testing using CLINITEX Microalbumin Reagent Strip (Siemens Healthcare GmbH), however, the POC testing yielded a high number of false positives (See **Text** **Box** 1) and was abandoned in favour of laboratory urinalysis. Albumin levels between 2.8 and 29.9 mg/mmol were considered *abnormal* (i.e. microalbuminuria), and ≥30 mg/mmol were deemed *highly abnormal* (i.e. macroalbuminuria). Participants with macroalbuminuria were referred to the University College London (UCL) National Amyloidosis Centre at Royal Free Hospital in North London for diagnostic assessment for AA amyloidosis.

Survey data were collected in Open Data Kit (https://opendatakit.org/) and transferred into Stata 15 (StataCorp: College Station, TX) for analysis.

All participants were provided with study information sheets and gave written consent prior to answering the questionnaire and providing a sample for urinalysis. Participants were reimbursed for their time with a £10 gift voucher. All methods were carried out in accordance with the relevant guidelines and regulations. This study has been reported against the STROBE reporting checklist for observational studies^14^.

**Figure 1:**
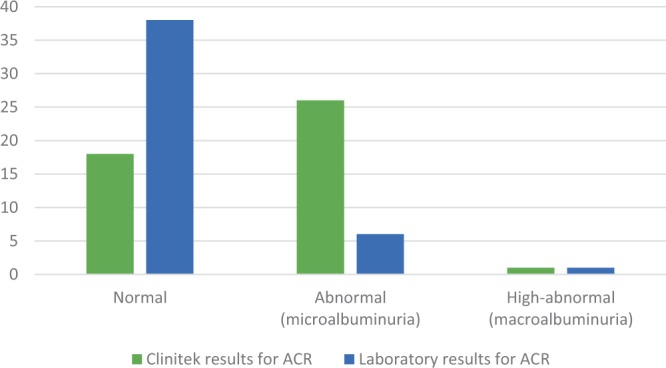
Discrepancy between CLINITEK and laboratory results for ACR.

Text Box 1 We initially used CLINITEK Microalbumin 2 Strips in a CLINITEK Status+ Analyser to determine the albumin-to-creatine ratio (ACR) of urine samples. CLINITEK Microalbumin 2 Strips were selected as they are specified for use with spontaneous urine samples and measure both albumin and creatinine values, enhancing the specificity of results. Despite manufacturer guidelines and reviews assuring adequate specificity and sensitivity for POC testing, we found a discrepancy in 20 of the 45 samples that were also sent for laboratory testing. While POC results indicated albuminuria in 58% of cases (26 micro and 1 macro), laboratory results for the same samples showed 13% with albuminuria (6 micro and 1 macro). (See Figure 1) Given this discrepancy we abandoned POC testing. We brought on an additional site with a laboratory pathway, and developed a collaboration with the UCLH Find & Treat Service outreach team, enabling us to recruit through homeless hostels via their outreach team and to use their private laboratory pathway.

The discrepancy between POC samples and laboratory samples is likely due to the highly concentrated urines of many participants. Concentrated urine has a very high creatinine level, which would have exceeded the microalbumin strip cut-off point – thus creating a false positive. Highly concentrated urines were also observed on handling, indicating inadequate water consumption and possible dehydration in this population. Aside from the health implications of dehydration, our findings indicate that semi-quantitative POC testing should be avoided or used with extreme caution with this population.

### Patient and public involvement

The survey was developed in collaboration with the Lambeth and Camden Drug User Forums. Members of the National Amyloidosis Centre Patient Network helped develop the participant information sheets. Groundswell, a local homelessness charity^15^, provided input to the study as well as peer support^16,17^ (See GRIPP2 Reporting Checklist **Appendix 1**).

### Sample size

We aimed to collect urine samples from 400 participants based on feasibility (rates of service attendance) and an estimated 5% prevalence of proteinuria (95% CI, 3–7%).

### Quantitative variables

The main outcomes of interest were any albuminuria; microalbuminuria (a biomarker for other health outcomes, such as cardiovascular disease) and macroalbuminuria (a biomarker for AA-amyloidosis). Exposures were chosen *a priori* as plausible factors in the causal chain and were based on the literature, our clinical experience, and our prior research involving PWID. Exposures include: injecting practices, location of injection sites, number of injection sites used, SSTI history, severity of past or current SSTI, housing status, overuse of acidifier (for dissolving drugs prior to injection), and self-reported lifetime diagnoses of health conditions (e.g. necrotising fasciitis, COPD, sepsis, endocarditis). Smoking status was added to the survey following commencement of data collection owing to the unexpectedly high prevalence of microalbuminuria, and is thus only available for 75% of the total sample, and 95% of the subsample of participants for whom we have laboratory results.

### Statistical analysis

Associations between potential risk factors and albuminuria were tested using crude logistic regressions. Bivariate associations found to have a significance of ≤0.1 were introduced into the multivariate logistic model to examine independent associations with albuminuria. We used a 95% confidence interval for the calculation of population point estimates of albuminuria. We further estimated a ratio of the prevalence of albuminuria in our PWID sample to the prevalence in the general population using data from the Health Survey for England 2016, which included urinalysis. We fit a poisson regression model on the combined data with albuminuria as the dependent variable and drug injection as the independent variable, adjusting for age group and sex. We then additionally adjusted for smoking status (current, former, or non-smoker).

### Ethics Approval

The study was approved by the NHS Health Research Authority (218143), the NHS Research Ethics Committee (17/LO/0872), and the London School of Hygiene & Tropical Medicine Observational Research Ethics Committee (12021–1). Participants gave informed consent to participate in the study.

## Results

Of the 455 participants who completed the survey, 442 consented to urinalysis. Three-hundred and sixteen laboratory urinalyses were returned. The 139 samples not included in the analysis were either tested using the POC CLINITEX Microalbumin Reagent Strips only (e.g. for services with no laboratory pathway) or they were rejected by the laboratory (e.g. the sample container was damaged). We compared the personal characteristics of those from whom we obtained a sample to those from whom we did not using the Mann–Whitney U test (for age) and Pearson χ-squared and found no significant difference between the two groups (See **Appendix 2**).

### Descriptive data

The majority of the 316 participants were white (73%) and male (75%), both their mean and median age was 46 years old (IQR 39–52). The average age at first injection drug use was 24 years (See Table 1). Of the 316 participants, 169 (54%) reported a diagnosis of HCV, 77 (24%) deep vein thrombosis (DVT), 45 (14%) chronic obstructive pulmonary disease (COPD), 27 (9%) kidney disease, and seven (2%) necrotising fasciitis. Seventeen (5%) participants reported receiving an HIV diagnosis (See Table 2).

**Table 1:** Participant Characteristics.

	N = 316	%
**Age**		
<35	44	13.9
35–44	85	26.9
45–54	137	43.4
>54	50	15.8
**Gender**		
Male	236	74.7
Female	80	25.3
**Stability of housing in previous 12 months**		
Stable (own place, rented)	136	43
Unstable (other people’s places, street homeless, hostel dwelling)	180	57
Ever been street homeless	242	76.6
**Ethnicity**		
White or White British	230	72.8
African, Caribbean or Black British	39	12.3
Asian or Asian British	7	2.2
Mixed ethnicity	21	6.7
Other	19	6

**Table 2:** Participant Lifetime Morbidities.

	N = 316	%
Hepatitis C	169	53.5
Deep Vein Thrombosis (DVT)	77	24.4
Arthritis	71	22.5
Hypertension	65	20.6
Sepsis	63	19.9
Chronic Obstructive Pulmonary Disease (COPD)	45	14.2
Hepatitis B	40	12.7
Liver cirrhosis	39	12.3
Kidney disease	27	8.5
Diabetes	18	5.7
Bone or joint infection	18	5.7
HIV	17	5.4
Endocarditis	14	4.4
Tuberculosis	13	4.1
Necrotising fasciitis	7	2.2
Lymphangitis	4	1.3

The majority of participants reported having a history of street homelessness (n = 242, 77%), with 57% (n = 180) currently living in unstable accommodation. The majority reported a typical injecting practice which included injecting one or more times per day (n = 227, 72%), and/or injection into the femoral vein (lifetime: n = 136, 43%, past 12 months: n = 62, 32%) (See Table 3). One-hundred and thirteen (39%) reported over-use of acidifier (defined as more than half a sachet of citric acid or vitamin C^6^) when preparing a £10 bag of heroin. Three hundred participants provided data on smoking status; 272 (91%) reported current or past smoking.

**Table 3:** Injection Practices.

	n	%
Ever injected psychoactive drugs for non-medical purposes^†^	316	100
Ever injected in arms	301	95.3
Ever injected in hands	234	74.1
Ever injected in legs	193	61.1
Ever injected in feet	177	56
Ever injected in groin	136	43
Ever injected in neck	119	37.7
Ever injected in other	46	14.6
Injected in the last 12 months^‡^	190	60.1
Heroin alone	136	71.6
Heroin and crack	134	70.5
Crack alone	57	30
Powder cocaine	22	11.6
Amphetamine	16	8.4
Ketamine	10	5.3
Other	6	3.2
Prescription diamorphine	12	6.3
Methadone	6	3.2
Mephedrone	6	3.2
Heroin with amphetamines	9	4.7
Steroids or other performance enhancing drugs	2	1.1

^†^In order from most commonly identified as ‘main bodysite injected’, to least.

^‡^In order from most commonly identified as ‘main drug injected’, to least.

### Outcome data

Of the 316 urinalysis results, sixty-one (19.3% [95% CI 15.3–24.0]) were positive for albuminuria with ≥2.8 mg/mmol albumin/creatinine ratio. Of these, 52 (16% [95% CI 12.8–21.0]) were classified as having microalbuminuria, and nine (3% [95% CI 1.5–5.4]) had macroalbuminuria. Men and women differed slightly but not significantly: 21.2% (95% CI 16.4–26.9) of men and 15% (95% CI 8.6–24.8) of women were found to have albuminuria. The prevalence of albuminuria generally increased with age: 13.6% (95% CI 6.1–27.7) in participants aged ≤35, and 21.4% (95% CI 16.1–27.9) in those aged ≥45, however this was not significant (p > 0.05). The nine participants with macroalbuminuria were referred to the UCL National Amyloidosis Centre and were offered Groundswell peer support (four declined to attend, three attended, and two died before their appointment).

### Main results

In a series of crude analyses the following associations were found with albuminuria (See Table 4 for associations): acidifier overuse (OR 1.77, p = 0.061), COPD (OR 2.15, p = 0.033), HCV (OR 1.86, p = 0.037), DVT (OR 1.69, p = 0.090), HIV (OR 3.18, p = 0.025), and injecting in three body sites (OR 2.49 p = 0.079). There was no significant association between SSTIs (OR 0.87, p = 0.643) or groin injecting (OR 1.36, p = 0.282).

**Table 4:** Associations Between Albuminuria and Risk Factors.

Variable	Albuminuria (micro/macro)		Crude	Adjusted*	Multiple adjusted**
	NO (%)	YES (%)	OR (95% CI)	AOR (95% CI)	AOR (95% CI)
**Reuse needles and syringes**					
No	79 (81%)	18 (19%)	1	—	—
Yes	176 (80%)	43 (20%)	1.07 (0.58–1.98)	—	—
**Reuse filters**					
No	109 (80%)	28 (20%)	1	—	—
Yes	140 (82%)	30 (18%)	0.83 (0.47–1.48)	—	—
**Injecting frequency per week**					
Once	30 (77%)	9 (23%)	1	—	—
2–3 times a week	28 (85%)	5 (15%)	0.60 (0.18–2.00)	—	—
4–6 times a week	13 (76%)	4 (24%)	1.03 (0.27–3.95)	—	—
Once a day	28 (80%)	7 (20%)	0.83 (0.27–2.54)	—	—
2–3 times a day	99 (84%)	19 (16%)	0.64 (0.26–1.56)	—	—
≥ Four times a day	57 (77%)	17 (23%)	0.99 (0.40–2.50)	—	—
**Current smoker**					
No	25 (89%)	3 (11%)	1	—	—
Yes	221 (81%)	51 (19%)	1.92 (0.56–6.63)	—	—
**Ever inject in hands**					
No	69 (84%)	13 (16%)	1	—	—
Yes	186 (79%)	48 (21%)	1.37 (0.70–2.69)	—	—
**Ever inject in arms**					
No	13 (87%)	2 (13%)	1	—	—
Yes	242 (80%)	59 (20%)	1.58 (0.35–7.23)	—	—
**Ever inject in feet**					
No	115 (83%)	24 (17%)	1	—	—
Yes	140 (79%)	37 (21%)	1.27 (0.72–2.24)	—	—
**Ever inject in legs**					
No	102 (83%)	21 (17%)	1	—	—
Yes	153 (79%)	40 (21%)	1.27 (0.71–2.28)	—	—
**Ever inject in groin**					
No	149 (83%)	31 (17%)	1	—	—
Yes	106 (78%)	30 (22%)	1.36 (0.78–2.38)	—	—
**Number of sites ever injected**					
One	56 (86%)	9 (14%)	1	1	1
Two	35 (83%)	7 (17%)	1.24 (0.42–3.65)	1.16 (0.40–3.35)	0.73 (0.18–2.91)
Three	25 (71%)	10 (29%)	2.49 (0.90–6.89)	2.08 (0.71–6.06)	3.01 (0.98–9.22)
Four or more	139 (80%)	35 (20%)	1.57 (0.71–3.48)	1.36 (0.61–3.00)	1.53 (0.64–3.64)
**Ever had SSTI**					
No	82 (80%)	20 (20%)	1	—	—
Yes	173 (81%)	41 (19%)	0.97 (0.54–1.76)	—	—
**Severity of infection**					
Mild/moderate	85 (80%)	21 (20%)	1	—	—
Severe	65 (80%)	16 (20%)	1.00 (0.48–2.06)	—	—
**Unstable housing**					
No	113 (83%)	23 (17%)	1	—	—
Yes	142 (79%)	38 (21%)	1.31 (0.74–2.33)	—	—
**Acidifier overuse**					
No	152 (85%)	27 (15%)	1	1	1
Yes	86 (76%)	27 (24%)	1.77 (0.97–3.21)	1.71 (0.90–3.25)	2.10 (1.04–4.22)
**Diagnoses of health condition**					
HIV					
No	245 (82%)	54 (18%)	1	1	1
Yes	10 (59%)	7 (41%)	3.18 (1.16–8.73)	3.16 (1.06–9.41)	4.11 (1.37–12.38)
HCV					
No	126 (86%)	21 (14%)	1	1	1
Yes	129 (76%)	40 (24%)	1.86 (1.04, 3.33)	1.72 (0.93–3.20)	2.07 (0.99–4.31)
DVT					
No	198 (83%)	41 (17%)	1	1	—
Yes	57 (74%)	20 (26%)	1.69 (0.92–3.12)	1.37 (0.70, 2.67)	—
COPD					
No	224 (83%)	47 (17%)	1	1	1
Yes	31 (69%)	14 (31%)	2.15 (1.06–4.36)	1.85 (0.84–4.06)	1.97 (0.86–4.50)

*Adjusted for age, gender, and smoking. **Stepwise model adjusted for: age, gender, smoking, number of injection sites, over-use of acidifier, HIV, HCV, COPD.

Based on these associations we performed logistic regression using listwise deletion of observations with missing smoking status and adjusted for *a priori* confounders (i.e. age, gender, and current smoking). We found higher odds of having albuminuria amongst those who: reported using more than half a sachet of acidifier per £10 of heroin (aOR 1.71 [95% CI 0.90–3.25]); had been previously diagnosed with COPD (aOR 1.85 [95% CI 0.84–4.06]), HCV (aOR 1.72 [95% CI 0.93–3.20]), HIV (aOR 3.16 [95% CI 1.06–9.41]), and injecting in three body sites (aOR 2.08 [95% CI 0.71–6.06]).

Further adjustment for other covariates suggested that the risk factor with the largest association with albuminuria was HIV (aOR 4.11 [95% CI 1.37–12.38]) followed by over-use of acidifier in injection preparation (aOR 2.10 [95% CI 1.04–4.22]).

In order to determine the age-adjusted prevalence ratio we compared our sample to the Health Survey for England data for 2016. Accounting for smoking status and stratified by age, we excluded the youngest (<25) and oldest (65+) age groups as they included small numbers of observations. The adjusted prevalence ratio suggests that albuminuria is twice as prevalent (1.97 [95% CI 1.49–2.60]) in our population compared to the general population in England.

## Discussion

### Statement of principal findings

This study sought to determine the prevalence of albuminuria amongst PWID in London. Previous studies have estimated that albuminuria in the general adult population in the UK is less than 10%^12,18,19^. Our findings suggest prevalence amongst PWID is twice that at 19% (15.3–24.0%). However, the age distribution in our sample differs markedly from that of the general adult population^18^. Adjusting for current smoking the prevalence ratio decreased to 1.65 (95% CI 1.17–2.31), suggesting that about one-third of the increased prevalence relates to smoking. Within our sample of PWID, overuse of acidifier and HIV were important predictors of albuminuria.

HIV is known to be associated with proteinuric renal lesions^20^. Additionally, we hypothesise that persistent overuse of acidifier in injection solution is precipitating venous sclerosis (owing to endothelial stress), thus complicating venous access and resulting in multiple venous injection attempts (and possible *accidental* subcutaneous injecting) and/or transition to *intentional* subcutaneous injecting^21^. Subcutaneous injecting introduces substances and/or bacteria directly into tissues which, in turn, causes persistent localised inflammation^1^. Alternatively, we cannot rule out the possibility that over-use of acidifier is directly causing local or systemic inflammation^6^. Chronic inflammation is strongly associated with cardiovascular and renal disease of which albuminuria is a biomarker^9^.

These data suggest that PWID are a high-risk group to develop cardiovascular and renal complications. A recent study of mortality among people who use heroin in South London found 2.8 times the risk of cardiovascular mortality compared to the general population^22^. If the associations (as observed in the general UK population) between albuminuria and future risk of cardiovascular disease are also true for PWID, one would expect the stroke risk in this population to be increased by 90% relative to a person with no albuminuria^23^, whilst the absolute risk of cardiovascular mortality increases by about 30% for a doubling of albuminuria^24^.

### Strengths and weaknesses of the study

Initially, urine sample testing was carried out using a POC testing machine; however, false positives were identified following confirmatory laboratory testing We believe this to be a result of high levels of dehydration amongst PWID. Our prevalence estimate may have been improved by testing twice (as per NICE guidelines); however, this would have been difficult given our study population^9^. The Health Survey for England albuminuria data were collected based on the same protocol used for the present study (i.e. a single sample) therefore increasing its comparability with our study population^18^.

The absence of a significant association between SSTIs and albuminuria was surprising as we anticipated SSTIs would be a key factor in activating the inflammatory cascade. It is possible that our participants underreported past SSTIs or that our sample was underpowered to draw out this association. Our study may also have been underpowered to detect other meaningful predictor variables. Fnally, we cannot rule out the possibility that HIV and excessive use of acidifier may be contributing to a systemic microvascular disorder.

### Unanswered questions and future research

Future studies should investigate long-term outcomes of PWID realted to cardiovascular and renal risk, though we appreciate the challenges of following up a difficult to capture population. Future trials should investigate whether cardiovascular and renal disease may be delayed by blood pressure lowering therapy or a poly-pill (i.e. a pill containing a combination of several medications) in those with albuminuria. In addition, studies could explore the reversibility of albuminuria through risk reduction and/or entry into substance use treatment (e.g. opioid agonist therapy). We encourage evaluations of the efficacy and cost-effectiveness of albuminuria screening amongst PWID.

## Conclusion

Albuminuria is twice as prevalent among PWID compared to the general population and may identify those with high cardiovascular and renal risk. Clinicians, such as general practitioners, should be aware of the risk and consider albuminuria testing of PWID. Finally, dehydration may be common amongst PWID and may affect the diagnostic accuracy of POC testing for albuminuria. We encourage careful interpretation of albuminuria tests among this population.

### Supplementary information

Supplementary information.

## Data Availability

All data generated or analysed during this study are included in this published article and its supplementary information files.

## APPENDIX 1: GRIPP2 Short Form

**Table:**

Section and topic	Item	Reported on page No
1: Aim	Report the aim of PPI in the study	3
2: Methods	Provide a clear description of the methods used for PPI in the study	3
3: Study results	Outcomes—Report the results of PPI in the study, including both positive and negative outcomes	5
4: Discussion and conclusions	Outcomes—Comment on the extent to which PPI influenced the study overall. Describe positive and negative effects.	n/a
5: Reflections/critical perspective	Comment critically on the study, reflecting on the things that went well and those that did not, so others can learn from this experience	n/a

## APPENDIX 2: Table comparing demographics for those with and without an ACR result

**Table:**

	ACR result		No ACR result		Pearson’s chi squared		Mann Whitney U	
	N = 316	%	N = 139	%	χ2 (df)	P value	z	P value
**Age**							1.3	0.194
**<**35	44	13.9	21	15.1				
35–44	85	26.9	45	32.4				
45–54	137	43.4	57	41				
**>**55	50	15.8	16	11.5				
**Gender**					0.04 (1)	0.846		
Male	236	74.7	105	75.5				
Female	80	25.3	34	24.5				
**Stability of housing in previous 12 months**					0.07 (1)	0.794		
Stable (own place, rented)	136	43	58	41.7				
Unstable (other people’s places, street homeless, hostel dwelling)	180	57	81	58.3				
**Ever been street homeless**	242	76.6	113	81.3	1.25 (1)	0.263		
**Ethnicity**					4.00 (4)	0.407		
White or White British	230	72.8	106	76.3				
African, Caribbean or Black British	39	12.3	11	7.9				
Asian or Asian British	7	2.2	5	3.6				
Mixed ethnicity	21	6.7	6	4.3				
Other	19	6	11	7.9				
